# Universities as Intermediary Organizations: Catalyzing the Construction of an Age-Friendly City in Hong Kong

**DOI:** 10.1093/geroni/igad016

**Published:** 2023-02-21

**Authors:** Cheryl Hiu-Kwan Chui, Shiyu Lu, On Fung Chan, Johnson Chun-Sing Cheung, Yingqi Guo, Yuqi Liu, Terry Y S Lum

**Affiliations:** Department of Social Work and Social Administration, The University of Hong Kong, Hong Kong, China; Department of Social and Behavioural Sciences, City University of Hong Kong, Hong Kong, China; Sau Po Centre on Ageing, The University of Hong Kong, Hong Kong, China; Department of Social Work and Social Administration, The University of Hong Kong, Hong Kong, China; Department of Social Work, Hong Kong Baptist University, Hong Kong, China; Department of Urban Planning, School of Architecture, South China University of Technology, Guangzhou, China; Department of Social Work and Social Administration, The University of Hong Kong, Hong Kong, China

**Keywords:** Civic engagement, Conceptual development, Organizational and institutional issues, Policy, Social networks

## Abstract

**Background and Objectives:**

The construction of an Age-Friendly City (AFC) requires active contribution from relevant interest groups including older adults, nonprofit organizations, and policy-makers. However, given that relevant interest groups may have limited resources, knowledge, and skills, as well as unique contextual factors, they often require help from intermediary organizations—actors that aim to build interest groups’ capabilities. Our objectives were to examine the functions of universities, as an example of intermediary organizations, in facilitating the construction of an AFC, and identify critical factors that enable intermediary organizations to perform their functions.

**Research Design and Methods:**

We conducted three focus groups and one individual interview with multiple interest groups including older adults and social workers from nonprofit organizations and local government involved in a 6-year citywide AFC project in Hong Kong. Participants were asked to share their views on the role of universities in relation to their own experiences and roles in the project. Data generated from the interviews were analyzed using thematic analysis.

**Results:**

Four themes pertinent to the functions of universities in facilitating development were identified: facilitating cross-sector collaborations, knowledge diffusion, interest-group building, and mediating divergent interests. We also found that neutrality and reputability are key characteristics for intermediary organizations to wield sufficient legitimacy to perform their functions efficiently.

**Discussion and Implications:**

Findings underscore the important yet overlooked role of intermediary organizations in bridging and mediating different interest groups to facilitate AFC development. We advance gerontological scholarship by providing insights into the theoretical mechanisms and practice implications for intermediary organizations in fostering an AFC.

**Translational Significance:** There is little understanding of the role of universities as intermediary organizations in facilitating the construction of an Age-Friendly City (AFC) in the East Asian context. By integrating gerontological and innovation literature, and by drawing from multiple interest-group interviews comprising older adults, social workers from nonprofit organizations, and an elected official to the local government involved in a 6-year citywide AFC project, we found that universities performed four key functions as intermediary organizations to facilitate AFC construction. This research will inform jurisdictions in leveraging neutral and reputable intermediary organizations to continue advancing the AFC agenda.

Since 2005, the World Health Organization (WHO) has made concerted efforts to promote the “Age-Friendly City” (AFC) framework to “encourage active aging by optimizing opportunities for health, participation, and security in order to enhance quality of life as people age” (WHO, 2007). The subsequent two decades have seen numerous AFC initiatives implemented worldwide. By 2021, over 1,114 cities and communities across 44 countries had joined the WHO Global Network for Age-Friendly Cities and Communities (GNAFCC; WHO, 2021).

Existing studies examining the process of developing an AFC have emphasized the imperatives to adopt multistakeholder or cross-sector approaches (Black & Oh, 2022; Colibaba et al., 2020; Garon et al., 2014), garner political commitment and practical support (Buffel & Handler, 2018), involve older adults in decision-making processes (Scharlach, 2011), establish steering committees to oversee AFC initiatives (Buffel et al., 2016), integrate the AFC concept into existing services (Plouffe & Kalache, 2011), and implement continuous AFC monitoring mechanisms (Chui et al., 2022).

Although these enablers and strategic approaches provide insight into how AFCs can be constructed effectively, the existing studies have been predominately conducted in the West (Aw et al., 2017). As detailed in the following section, these findings may not be directly replicable in non-Western, quasi-democratic regions such as East Asia, where socioeconomic and political factors diverge (Chui et al., 2023). These divergences create unique challenges to facilitate an AFC. A central argument we put forth is the lack of focus in existing gerontological literature in integrating the concept of intermediaries derived primarily from innovation literature to advance our understanding of organizations that are able to overcome these challenges and to facilitate the coordination and smooth functioning of the construction of an AFC. Indeed, the role of *intermediary organizations* in AFC construction constitutes an important yet overlooked dimension in gerontological literature. Intermediary organizations, a term used in innovation literature (Hernández-Chea et al., 2021), refer to organizations that mediate the relationship between two or more social actors within particular ecosystems (van der Meulen et al., 2005). Intermediary organizations and their roles in facilitating knowledge exchange, industry capacity-building, and policy change have received growing attention (Blix Germundsson et al., 2020). However, a theoretical understanding of how intermediary organizations influence and facilitate AFC construction in non-Western, quasi-democratic settings is still lacking. In this study, we positioned universities as a type of intermediary organizations (Cameron, 2012). As will be shown later, universities played a unique role in facilitating AFC construction in Hong Kong.

This study situates itself in the intersection between gerontological and innovation literature. Drawing from interviews from multiple interest groups comprising older adults, social workers, and local government officials, we examine the following research questions: (1) What are the functions of universities, as an example of intermediary organizations, in facilitating the construction of an AFC in a non-Western, quasi-democratic context? (2) What organizational characteristics enable universities to perform these functions effectively in AFC construction? Findings carry theoretical and practical implications on gerontological literature in advancing understanding and optimizing the functions of intermediary organizations in AFC construction.

## Barriers to Achieving AFC in Non-Western, Quasi-Democratic Societies

The development of AFC is a highly contextualized and dynamic undertaking (Buckner et al., 2019). However, the critical enabling factors in constructing an AFC were predominately generated from the West (Suriastini et al., 2019). For example, although studies have emphasized cross-sector collaborations (Black & Oh, 2022; Greenfield et al., 2015; Pestine-Stevens & Greenfield, 2022), studies in East Asia found that care services remain fragmented despite the efforts to foster better coordination (He & Tang, 2021; Lau et al., 2018). Barriers include competition and mistrust among professionals (e.g., between medical and social care professionals), between private and public sectors (Lau et al., 2018), limited institutional capacity of nonprofit organizations (NPOs), and resource fragmentation among different interest groups (Lam, 2021).

Similarly, literature has long recognized the importance of involving older adults in the decision-making processes related to AFC. An example of this is engaging senior clubs and associations to define and prioritize key domain areas that will inform subsequent AFC interventions and resource allocations (Garon et al., 2014). This participatory approach differs from a professionally led approach, whereby professionals such as health and social workers define the needs of older adults, an approach typical in East Asia (Cheng et al., 2013). East Asian governance models also diverge from the West, in that policy-making processes are largely impervious to parties outside the policy-making system, and typically occur in a top-down manner, albeit to different degrees (Chao & Huang, 2016; Lee et al., 2013). These factors leave little room for older adults’ participation in decision-making processes.

Furthermore, although governments across East Asia have endorsed the AFC agenda, there is scant evidence that conceptual and technical knowledge of AFCs generated at global levels has been successfully imported and translated into local aging services (Sun et al., 2017). Effective policy-making and implementation require policy-makers to make informed, evidence-based decisions (Oxman et al., 2009). This appears to be lacking given that no systematic assessment or monitoring mechanisms have been implemented to evaluate age friendliness in cities such as Hong Kong (Chui et al., 2022). Without such evidence, it would be difficult for policy-makers to make informed decisions for AFC construction.

In sum, although enabling factors have been extensively discussed in gerontological literature, the socioeconomic and political configurations characteristic of many East Asian societies present unique challenges for AFC development, rendering the generalizability and applicability of Western-informed strategic approaches toward AFC construction (e.g., heavy involvement of older adults, cross-sector collaborations) questionable. Moreover, existing literature tended to focus on describing the nature of cross-sector collaborations and highlighting its importance (Black & Oh, 2022; Colibaba et al., 2020), thus neglecting the role that intermediary organizations play specifically.

### Intermediary Organizations

Intermediary organizations can take diverse forms (Knight & Lyall, 2013). These forms may include universities, think tanks, industry associations, research centers, and independent consultants (Blix Germundsson et al., 2020). Intermediary organizations operate within specific “ecosystems”—the entire environment characterized by a multiplicity of resources (such as financial, human, social/political, and intellectual capital), actors, relationships, interactions, and conditions (such as policy and economic and social conditions) that either enable or impede a certain cause (Cameron, 2012). In the context of technology innovation ecosystem, for example, studies have shown that intermediaries have a powerful influence as “change agents” in influencing the speed of technology diffusion and uptake of new products and services (Watkins et al., 2015) and policy-making (Kivimaa et al., 2020).

Typically, intermediary organizations undertake problem definition and articulation within a particular ecosystem, connect relevant actor groups, build and manage networks, translate information between different actors (e.g., across people, organizations, industries), consolidate and advocate specific interests, and facilitate knowledge exchange and competences among different agents, who themselves have different languages, organizational cultures, decision-making horizons, and systems of incentives (Caloffi et al., 2015; Kivimaa et al., 2020).

Borrowing the ecosystem framework, we argue that AFC construction constitutes an ecosystem of its own (Fulmer et al., 2020; Wetle, 2020). An age-friendly ecosystem is the “comprehensive, collectively built, and ever-expanding platform whose goal is to improve older adults” quality of life through enhanced, collective impact’ (Batchelor, 2021). Echoing the notion that AFC domains are interconnected (Buffel & Handler, 2018), an AFC ecosystem recognizes that there are complex, interdependent, and dynamic structures and processes that affect the development of an AFC. Akin to other ecosystems, the AFC ecosystem requires substantial resources, coordination, and harmonization of relevant interest groups such as government agencies, NPOs, older adults, and businesses.

As aforementioned, the unique socioeconomic and political configurations characteristic of East Asian societies present both risks (barriers) and opportunities for the promotion of AFCs. These contextual factors leave room for intermediary organizations to take on important roles and functions in enhancing the AFC ecosystem, and in supporting, brokering, and addressing challenges and divergent interests across multiple interest groups. One study on innovative pathways to building age-friendly homes in Europe highlighted the importance of “support structures”—defined as an overarching organizational element that supports experimental activities—in facilitating the continent’s transition toward age-friendly housing (Sengers & Peine, 2021). An example of “support structures” presented in this study was intermediary organizations, and their role in developing and supporting experiments around age-friendly housing that are not yet mainstream. Sengers and Peine (2021) further argued for the need to examine the neglected role of these “support structures” in facilitating change. Indeed, there has been little research on examining the role of intermediary organizations in facilitating the process of AFC construction. Furthermore, studies in the field of innovation have cautioned that despite the growing prominence of intermediary organizations in facilitating ecosystem change, it is not immediately clear what organizational characteristics are best suited to play an intermediary role (Caloffi et al., 2015).

Our study fills these gaps by unraveling the functions of universities, as one of many possible types of intermediary organizations, in constructing an AFC. We also shed light on specific qualities of universities to explain how they can wield influence within an AFC ecosystem.

## Hong Kong: The Jockey Club Age-Friendly City Project as a Case Illustration

Hong Kong is a highly urbanized city housing approximately 7.4 million people. The proportion of older adults aged 65 and over is projected to increase from 1.32 million (18.4% of the population) in 2019, to approximately 2.52 million (33.3%) in 2039 (Census and Statistics Department, 2021). The Jockey Club Age-Friendly City Project (JCAFC; https://www.jcafc.hk/en/index.html) constitutes the city’s largest AFC intervention. Funded by the Hong Kong Jockey Club Charities Trust, this 6-year project has three overarching objectives: (1) to assess the age friendliness of each district; (2) to recommend an AFC framework for districts to undertake; and (3) to raise public awareness and encourage community participation in building an AFC. The project was implemented in two phases. Phase I took place between 2015 and 2018 covering eight districts whereas Phase II took place from 2017 to 2021 and covered all 18 districts of Hong Kong.

The implementation of JCAFC comprised a four-stage model (Chui et al., 2022). Stage 1 conducted a district-wide baseline assessment of the AFC; Stage 2 implemented community AFC interventions in response to the findings from Stage 1. These interventions were implemented by social workers in nonprofit centers for older people across Hong Kong. Stage 3 established future AFC policy action plans and Stage 4 conducted a final district-wide assessment.

A key feature of JCAFC was the heavy emphasis it put on four universities as intermediary organizations in facilitating project implementation. Universities have been regarded as intermediary organizations in literature (Cameron, 2012). Recent studies on AFC also found local universities also performed an important linking function across different interest groups, which was critical to establishing collaborations (Pestine-Stevens & Greenfield, 2022). All four universities performed several tasks and activities in the JCAFC (Chui et al., 2022). First, universities conducted a district-wide baseline assessment with older adults to assess their overall age friendliness using the 52-item scale based on the WHO’s Age-Friendly City Framework and Guidelines (2007) and focus groups. Second, universities elicited NPOs’ support to participate in the project. Based on the baseline findings, social workers in these NPOs created and implemented AFC community interventions to address deficit areas and universities evaluated their effectiveness. Third, universities liaised with local governments, the purposes of which were to introduce the AFC framework, share research findings and establish AFC-related policy action plans and assist the government in submitting the best practices to GNAFCC. Finally, universities implemented a series of “AFC ambassador training schemes.” These ambassador training schemes were co-implemented with NPOs to equip older adults with the knowledge of AFC. Older adult ambassadors were then tasked to observe and monitor social and physical environmental domains in their districts and advocate the AFC agenda. Given the relatively low levels of civic engagement extant among older adults (Chui et al., 2019), universities and NPOs alike proactively reached out to community-dwelling older adults to participate in the project.

At the completion of the JCAFC in 2021, 140 AFC district-based community interventions involving 80 NPOs were completed, and more than 2,500 older adults became AFC ambassadors. All Hong Kong’s 18 districts were admitted to the GNAFCC and local government across all districts endorsed the recommended AFC policy action plans. A prior evaluation study on this project also showed that older adults’ perceived age friendliness of all eight domains had increased significantly (Chui et al., 2022).

## Method

This qualitative study used focus groups and individual interviews to address the research questions. The use of focus groups or individual interviews during the data collection process depended on the availability of participants. Participants were recruited through purposive sampling from people who were actively involved in the JCAFC Project. They included two focus groups with 15 older adults who were AFC ambassadors, and had actively assisted in the implementation of AFC community interventions; one focus group with three social workers from nonprofit centers that work exclusively with older people who implemented AFC community interventions during the project period to improve age friendliness in their districts; and an individual interview with one elected politician in local government who worked closely with the research team to endorse research findings, approve funding of AFC community interventions, establish future AFC policy action plans, and submit best practice examples to GNAFCC. Table 1 summarizes the type of methodology employed in the data collection process. We stopped focus group interviews when data saturation was achieved (Fusch & Ness, 2015). All interviews were conducted between July and September 2022, conducted in Cantonese, and were audio-recorded.

**Table 1.** Methodology Employed in Data Collection Process

Type of qualitative research method	Venue	Participants (*N* = 19)
Focus group 1	Nonprofit center for older people in Hong Kong	Older adult participants in JCAFC (*n* = 7)
Focus group 2	Nonprofit center for older people in Hong Kong	Older adult participants in JCAFC (*n* = 8)
Focus group 3	Virtual (in Hong Kong)	Social workers in nonprofit centers for older people (*n* = 3) who were involved in JCAFC
Individual interview	Virtual (in Hong Kong)	Elected official to local government (*n* = 1)

*Note*: JCAFC = Jockey Club Age-Friendly City Project.

All focus group and individual interviews utilized a semistructured interview guide. The set of questions asked was the same across all relevant interest-group groups, with slight moderations in wording (see Supplementary Material). In each interview, all participants—be they older adult participants, social workers, and the elected official—were asked to recall their experience in the JCAFC and to explain their respective roles in the project. Participants were then asked to share their perceptions of the universities involved in the JCAFC and to elaborate their answers by providing examples from their own experiences of interacting with universities to identify the functions of universities, as an example of intermediary organizations, and how such functions facilitated (or impeded) their respective work within JCAFC. They were further asked to reflect on and identify qualities or characteristics of universities that have enabled them to perform those functions. Focus groups lasted approximately 1 hr, and were held in NPOs convenient to participants. The demographic characteristics of older adult participants in the two focus groups were also collected. Table 2 illustrates older adult participants’ demographic details. At the end of the interviews, older adult participants were given a store coupon of HK$50 to thank them for their participation.

**Table 2.** Demographic Characteristics of Older Adult Participants (*n* = 15)

Characteristics	*N* (%)	*M* (*SD*)
Gender		
Male	5 (33.3)	
Female	10 (66.7)	
Age		65.9 (13.7)
Education level		
Primary or below	3 (20.0)	
Secondary education	9 (60.0)	
Postsecondary or above	3 (20.0)	
Marital status		
Married	5 (33.3)	
Others	10 (66.7)	
Years of living in the community		
30 years or less	6 (40.0)	
31–50 years	4 (26.7)	
51 years or more	5 (33.3)	
Work status		
Employed	3 (20.0)	
Retired	9 (60.0)	
Homemaker	3 (20.0)	
Type of housing		
Public rental housing	7 (46.7)	
Home ownership scheme flats	3 (20.0)	
Private housing	4 (26.7)	
Others	1 (6.7)	
Monthly income		
HKD 5,999 or below	8 (53.3)	
HKD 6,000–14,999	1 (6.7)	
HKD 15,000–29,999	5 (33.3)	
HKD 30,000 or above	1 (6.7)	

*Notes*: HKD = Hong Kong dollar; *SD* = standard deviation.

Interviews with social workers and the elected local government official lasted approximately 45 min and were conducted virtually. However, we did not present monetary coupons to social worker participants or the elected official. We also did not collect demographic details of social workers or the elected official as our focus was on understanding their professional roles in JCAFC. The study was approved by the Research Ethics Committee of the University of Hong Kong.

### Data Analysis

The audio records of focus groups and individual interviews were transcribed verbatim and analyzed using thematic analysis (Braun & Clarke, 2012). The authors of this manuscript sought to identify common themes across all three interest groups. This comprised several stages. In Stage 1, we familiarized ourselves with the data by reading the transcripts thoroughly. In Stage 2, an initial list of codes relevant to our research objective was generated. At this stage, quotes describing participants’ views, opinions, and experiences in relation to the universities’ function in the project were highlighted. A sample quote that was highlighted during this coding process was from a social worker participant: “it was very useful for [universities] to introduce [their NPO] to relevant government departments or other professionals.” This was initially coded as “bridging networks.” In Stage 3, preliminary themes were identified by categorizing the initial codes. Following the previous example, when an emergent pattern indicated that both social work and older adult participants found universities helpful in linking them to relevant government departments in advancing the AFC agenda, we classified and categorized these codes into the emergent theme “facilitating cross-sector collaboration.” We compared the codes and these emergent themes within the research team to ensure reliability (Thomas, 2006). Stage 4 entailed the review and refinement of emergent themes. Stages 5 and 6 involved finalizing the naming of the themes and reporting.

One methodological concern is that we were cognizant that our own identities as university researchers and our roles in the JCAFC could affect the data collection process and analysis (Jootun et al., 2009). We had prior interactions with older adult participants, for example, the research team had trained some of the older adult participants in ambassador training schemes, which could increase our susceptibility to subjectivity or could affect participants’ responses. Hence, we took steps to minimize social desirability and improve data reliability (Jootun et al., 2009). For example, we assured participants that any views on universities, positive or negative, would not produce any adverse outcomes. Previous studies on qualitative methodologies have suggested that examples of common cues suggesting desirability tendencies are excessive praise or nervous facial expressions (Bergen & Labonté, 2020). The researchers were aware of these cues prior to conducting data collection. Thus, strategies such as self-disclosure, displays of respect, constant assurance, and clarification on how the data would be used to improve the research team’s understanding of their own roles in AFC construction were deployed. Likewise, we assured both social worker and government official participants that their views would have no adverse consequences. Because data collection took place after the end of the JCAFC, any evaluation work conducted by universities on AFC community interventions or local districts’ age friendliness had already been completed. There were also no ongoing collaborative projects or evaluations between universities, the local government, and the nonprofits at the time of data collection.

## Results

### Functions of Universities in AFC Construction

Our first research question was to identify the functions of universities, as an example of intermediary organizations, in facilitating AFC construction. Our analysis identified four key themes relating to the functions of universities in facilitating AFC construction: (1) facilitating cross-sector collaboration; (2) knowledge diffusion; (3) interest-group capacity-building; and (4) mediating divergent interests. A summary description of the functions of universities is presented in Table 3.

**Table 3.** Functions of Universities as Intermediary Organizations in AFC Construction

Functions	Description	Selected example(s)
Facilitating cross-sector collaboration	Universities was to act as a bridge *within* and *across* sectors by facilitating collaborations	In an AFC community-based intervention, the responsible NPO sought to improve residents’ knowledge of and capacity to address the rising prevalence of dementia within the district. However, this NPO primarily provided social services and did not have the medical knowledge and skills to conduct professional assessment and training. As a result, one participating university bridged another NPO specializing in dementia care in the health care sector with the other NPO in the social care sector
Knowledge diffusion	Universities broke down information asymmetry for different interest groups	Given that many older adults in Hong Kong have received limited formal education, universities used innovative methods such as photo-voice to make global information on AFC accessible to older adults and to enable them to easily understand, apply, and promote the AFC concept
Interest-group capacity-building	Universities enhanced interest groups’ capacities in their respective roles in building an AFC	A social worker remarked that they were able to use the empirical evidence generated from universities as a form of capital that enhanced their capability in advocating for change with the government
Mediating divergent interests	Different interest groups have their own respective agenda. For instance, although older adults have a wide range of needs, and NPOs may struggle to fulfill older adults’ needs under considerable resource constraints. Universities served to mediate these divergent interests by establishing common ground and consensus	A social worker explained that the government had their own priorities or agenda that did not always align with their own. In these circumstances, universities functioned as a “broker” to assist both parties navigate through their different interests regarding the construction of an age-friendly city

*Notes*: AFC = age-friendly community; NPO = nonprofit organization.

#### Facilitating cross-sector collaboration

Echoing studies on intermediary organizations (Barraket, 2020; Ho & Yoon, 2022), a key function of universities was to act as a bridge *within* and *across* sectors by facilitating collaborations. Universities had long held prior relations with NPOs specializing in aging services. By leveraging and mobilizing these existing networks, universities were able to initiate contact across sectors within the JCAFC and facilitate the formation of cross-sector collaboration between NPOs and other interest groups in a society traditionally characterized by intersector fragmentation (Lau et al., 2018).

For example, in an AFC community-based intervention, the responsible NPO sought to improve residents’ knowledge of and capacity to address the rising prevalence of dementia within the district. However, this NPO primarily provided social services and did not have the medical knowledge and skills to conduct professional assessment and training. As a result, one participating university bridged another NPO specializing in dementia care in the health care sector with the other NPO in the social care sector. Eventually, these two NPOs collaborated and together implemented the AFC intervention (Sau Po Centre on Ageing, 2021).

Likewise, in reference to another AFC community-based intervention producing a short film documentary on the district’s age friendliness, the social worker explained that universities were able to bridge professionals and students specializing in film production to collaborate with older adults. The university did so by recruiting interested students to join the project and by introducing production professionals to the NPO. As one social worker illustrated:

I think it was very useful for you (universities) to introduce us to other professionals when we implemented the micro-film project … our work is very localised and sort of bounded by our service or [geographic] scope, so it’s useful for universities since they have many connections. (Social worker #3)

Similarly, universities enabled older adults to reach relevant government officials which would have otherwise been difficult to achieve. Although this does not denote a formal collaboration between older adults and the government per se, a key function of intermediary organizations is to transcend boundaries and draw together initial dialogue (Bryson et al., 2006, 2015), especially in contexts where channels for civic participation are limited. As one participant explained:

[Universities] are very helpful because they can help us reach the relevant government department about our concerns. It would be difficult for the average older adult to reach the appropriate ears given that we have so many concerns about our communities and so many government departments may be involved. (Older adult participant)

#### Knowledge diffusion

The second key function that universities performed was knowledge diffusion. We found that universities broke down information asymmetry for older adults, many of whom were unaware of the AFC concept, through *ambassador training schemes.* Given that older adults had had fewer opportunities in attaining formal education in Hong Kong, universities used innovative methods such as photo-voice to make global information on AFC accessible to older adults and to enable them to easily understand, apply, and promote the AFC concept (Chui et al., 2019). As one participant explained:

Universities were very helpful because they taught us some of the newest concepts on AFC. There is too much information out there and we may get very confused, so universities helped us decipher this information and explain to us those big ideas and concepts … Otherwise we would be lost. (Older adult participant)

Similarly, as most social workers are overwhelmed with frontline work, they have little time to systematically sift through and consolidate the latest global developments on AFCs. In this vein, universities provide access and impart new knowledge relevant to local aging services. As participants explain:

We have frontline experiences and can deal with clients on a daily basis. But we have difficulty in grasping the “technical” or “conceptual” level [of AFCs] … like those from the World Health Organization. We understand baseline assessment was important so we can assess the age-friendliness of the community, but how are we supposed to do that? What kind of information are we supposed to collect, who should I ask? How do we measure [age-friendliness]? It was so important that universities were able to translate these abstract concepts into actual measures for us. (Social worker #1)

#### Interest-group capacity-building

Our third theme revealed that universities enhanced interest groups’ capacities in their respective roles in building an AFC. For example, a social worker remarked that they were able to use the empirical evidence generated from universities as a form of capital that enhanced their capability in advocating for change with the government:

The research findings that [universities] generated are helpful to us when we want to speak to the government … when we point out community deficiencies, it’s usually just unsystematic…the market here doesn’t have barrier-free facilities, the bus station doesn’t have chairs there ... It’s much more effective if I go and talk to them with data to back it up. (Social worker #1)

Social workers also explained that the knowledge gleaned from universities has enabled them to strategically incorporate AFC elements into existing services:

The research conducted by universities helped us reflect on what is missing in [older adult] services. For example, one of the survey findings was about how end-of-life care was missing—so we took these findings and incorporated them to enhance our service scope. (Social worker #3)

Similarly, social workers felt that their professional capacity was enhanced by learning how universities systematically manage and present findings in a way that corresponds to the WHO’s requirements for the admission to GNAFCC:

We wanted to be admitted to [GNAFCC] for a long time. But we didn’t have the capacity to sort out all that information and consolidate our experience as a [best practice] ... or how to present or write about our experiences … so the universities really helped us complete that process. I also learned a lot. (Social worker #3)

A government official also alluded to the role of universities in building local government capacity, especially in using research to identify specific AFC domains to be addressed as policy action plans:

The research helped guide some of our policymaking decisions. We’re not able to do it ourselves obviously. But the research was very useful—we can use the data to talk to other government departments … It also helped us make better decisions about what is needed in our community. (Government official participant)

#### Mediating divergent interests

The final emergent theme was mediating divergent interests. We found that participants were aware that priorities could differ from one interest group to another, as the government may have their own agenda, whereas older adults have a wide range of needs, and NPOs may struggle to fulfill older adults’ needs under considerable resource constraints. Universities served to mediate these divergent interests by establishing common ground and consensus. As participants explained:

We [older adults] have many concerns, but there are many divergent interests in society ... So, universities can at least provide us with some direction … some guidelines about priorities and explain to us what [will work] or what others think about our proposals. (Older adult participant)

We actually need universities to lead us through the project … teach us how to identify and weigh different options and areas … I mean, this older adult wants the elevator to be built, that one wants more housing modification support … but young people may not think that way … so universities help guide us through the different interests. (Older adult participant)

A social worker also felt the government had their own priorities or agenda that did not always align with their own. In these circumstances, universities functioned as a “broker” to assist both parties to navigate through their different interests:

It was good that universities were there when we voiced out our concerns to the district council. In a way, [their] presence gave them additional pressure to be more responsive to our requests … I learned a lot just by observing how [universities] presented the findings in a way that make [the government] see it is beneficial to them too. (Social worker #3)

### Achieving Legitimacy: Neutrality and Reputability as Key Characteristics of Universities

Our second research question was to identify characteristics or qualities that enable universities to perform their functions effectively in the construction of an AFC. Our analysis revealed two emergent themes: neutrality and reputability.

#### Neutrality

Participants unanimously agreed that neutrality was a critical attribute of universities enabling them to garner sufficient social legitimacy from interest groups within the AFC ecosystem. Because of the universities’ scientific approach to researching and informing the AFC agenda by way of collecting and analyzing empirical data, results from universities in examining local districts’ age friendliness were accorded legitimacy and credibility. They were, therefore, in a good position to garner support from different interest groups. As two social workers explained:

For sure, because universities are neutral—they look at the data in a scientific way. When we talk to the government, they will always think we are biased, that we have our own agenda, but because there is actual scientific evidence, this makes the situation much more neutral and easier for people to digest. (Social worker #1)

The government perceives us (NPOs) as “troublemakers” or that we are just there to launch complaints. If that’s the case, how will they ever treat our raised concerns fairly? That’s why there must be a neutral third party to act as a middleman to help negotiate and manage different parties’ expectations. (Social worker #3)

Similarly, an older adult participant espoused that legitimacy was entrusted to universities because they make observations and corresponding recommendations based on scientific empirical data, and collect views from different parties (instead of furthering only one interest group’s agenda). These two aspects render universities more neutral:

It’s because universities have a more neutral stance. They have the research and evidence to back up what they say … they hear things and views from everyone … us older adults, the government, or others but it seems they are more neutral and can treat the whole thing (the development of AFC) more fairly. (Older adult participant)

#### Reputability

Older adult participants indicated that the universities’ authoritative and reputable standing in society was another key characteristic that garnered legitimacy and credibility:

It’s the university’s prestigious reputation that makes the project very appealing to me. Universities have research to support, and the universities’ position in society is much higher than ours [older adults] … like with those ambassador training schemes you run, the endorsement from universities made it more convincing and legitimate. (Older adult participant)

Universities have a good reputation in society … people trust what [they] say. A lot of my peers joined the JCAFC because we knew the university was involved as a major player. (Older adult participant)

Similarly, one social worker explained that universities carry with them a certain prestige and authority in society that appeal to older adult participants, many of whom had few opportunities to receive formal or higher education due to historical and socioeconomic circumstances (Cheung et al., 2001).

It’s different when older adults receive training from NPOs rather than universities. They will feel the training [from universities] are of a higher level. Many of them have never even entered universities so they may think that whatever knowledge is imparted is more cogent. It motivates them more. (Social worker #3)

## Discussion and Conclusion

There has been little understanding of the role of intermediary organizations in facilitating the construction of AFC in quasi-democratic societies. Likewise, there is a limited understanding of the organizations best suited to subsume the role of intermediaries (Caloffi et al., 2015). Drawing on interviews from multiple interest groups, this study fills these research gaps and found that universities, as an example of intermediary organizations, performed four key functions: facilitating cross-sector collaboration, knowledge diffusion, interest-group capacity-building, and mediating divergent interests. We advance gerontological scholarship by providing empirical evidence on the critical role that universities play in an AFC ecosystem in a non-Western, quasi-democratic context. We also illustrate that neutrality and reputability are key characteristics embodied by universities, which help to explain why, in our study context, universities were able to garner sufficient legitimacy across multiple interest groups to perform their functions effectively (Bryson et al., 2006). Several additional insights can be generated from this study.

First, echoing existing studies in innovation literature and its emphasis on intermediary organizations in social capital and network-building (Barraket, 2020), universities were seen to subsume a “linking” function in that they bridge relevant actors within the AFC ecosystem to create a greater impact, such as implementing novel community-based AFC interventions. This finding was echoed more recently by a study conducted by Pestine-Stevens and Greenfield (2022), who also highlighted the function of universities as a “convener” and “connector,” and whose involvement improved the visibility of AFC initiatives. Furthermore, the “linking” function is essential, especially in contexts where intersectoral fragmentation is rife (Lau et al., 2018), and aligns with existing gerontological scholarship emphasizing the need to develop cross-sector approaches toward constructing an AFC (Buffel & Handler, 2018; Garon et al., 2014).

Beyond the “linking” function, knowledge diffusion is another key function performed by universities. As universities are knowledge producers, privy to cutting-edge global knowledge and concepts in relation to AFC, they were able to function as “knowledge brokers” (Knight & Lyall, 2013), importing global knowledge to local contexts and disseminating this knowledge to interest groups such as older adults, NPOs, and local government. In this light, universities appear to be able to overcome what Sun and associates (2017) describe as the lack of global–local articulation of AFC in East Asia.

Second, universities enhance interest groups’ capabilities in contributing toward an AFC. As our findings show, universities, as an example of intermediary organizations, were able to build NPOs’ professional capacity in advocacy and service planning, enable local governments to make better evidence-informed decisions, and enhance older adults’ competence in translating knowledge into practice through ambassador training schemes. To this end, universities contribute to ecosystem building by enhancing the capability of relevant interest groups. This finding is in line with emerging evidence that intermediary organizations are “system builders” (Hernández-Chea et al., 2021; Stam, 2015).

Third, attesting to the notion that the construction of an AFC is a highly contextualized phenomenon (Buckner et al., 2019), we provide insight into the nuances within an AFC ecosystem in a quasi-democratic society, where centralized decision-making processes are dominant, aging services are fragmented, and older adults have few opportunities for civic participation. These unique dynamics create barriers to replicate enabling factors mentioned in prior literature generated predominately from the West (Plouffe & Kalache, 2011; Scharlach, 2011; van Hoof et al., 2021), but also presented a unique opportunity for universities to overcome these barriers by facilitating dialogue and cross-sector collaborations, and mediating divergent interests across multiple interest groups within the AFC ecosystem. This finding echoes emerging evidence that intermediary organizations influence collaboration dynamics in innovation ecosystems (Stam, 2015).

Fourth, we highlight the importance of neutrality and reputability in enabling universities to be effective intermediary organizations (Bryson et al., 2006) that can exert considerable influence in the AFC ecosystem. It is plausible that engaging universities may be strategically advantageous as East Asian cultures value teachers’ authority and scholars have traditionally wielded considerable merit and legitimacy in society (Marginson, 2011). However, universities need not be the only intermediary organizations in an AFC ecosystem. Scholars have recognized that different ecosystems may require different organizations to mediate relationships between interest groups (Cameron, 2012). Our findings suggest that insofar as intermediary organizations embody neutrality and reputability, they can contribute to the AFC ecosystem. For example, reputable think tanks and industry associations may play the roles of intermediary organizations. This echoes existing innovation studies that illustrate the use of incubators (intermediary organizations) as a key intervention approach as they act as neutral coordinators, aligning the various interests and logic of actors within a particular ecosystem (Hernández-Chea et al., 2021). Whether other types of intermediary organizations can carry out similar functions as effectively as universities in AFC construction remain underexplored and warrants further investigation.

Finally, despite the critical function that universities appear to play in facilitating an AFC, they are not without limitations of their own. For instance, any continuing role that universities may play in addressing the long-term sustainability of the AFC agenda remains underexplored. Following the completion of the JCAFC where funding had ceased, the degree of contribution shifted from universities to civil society (e.g., nonprofit centers for older people), older adults themselves, and the government. Continuing dialogue, and to a large extent collaboration, among these three interest groups will require continuous political will, commitment, competence, and resources. These factors may shift over time. Future studies could examine what roles universities or other types of intermediary organizations may play in the long-term sustainability of the AFC ecosystem.

Our findings are relevant to other East Asian jurisdictions characterized by similar socioeconomic and political configurations. In examining the process of constructing an AFC, gerontological scholars may benefit from integrating innovation literature in unearthing the role of intermediary organizations to facilitate this highly contextualized and complex process. Policy-makers or practitioners aimed at developing an AFC in their respective communities may also benefit from our study, through enlisting the involvement of intermediary organizations to mobilize networks and resources to enhance the implementation effectiveness of AFC initiatives.

### Limitations

This study has several limitations, which carry implications for future research. First, despite seeking views from multiple interest groups, questions were centered around the usefulness and functions of universities as intermediary organizations in constructing an AFC. Negative views on the role of universities did not emerge from our findings. Existing literature, however, does caution toward the over-romanticization of intermediary organizations (Cameron, 2012). This leaves room for future research in examining the limitations of intermediary organizations in facilitating the process of AFC development. Second, because a change in administration had occurred during the research period resulting from the social unrest in 2019 (Lee, 2020), we were unable to contact those who were no longer in the office whereas those who remained were reluctant to be interviewed. Nevertheless, the one elected official we interviewed was present during the entire 6 years of JCAFC. Although findings generated from this one interview with the elected official shed light on how universities’ work affected government’s decision-making processes, we acknowledge that such findings are limited in the generalizability in understanding the government’s view on intermediary organizations. As the political system continues to evolve in Hong Kong, it may be worthwhile to examine the role of the government in facilitating an AFC in the future. Third, although the findings show that universities played a prominent role in the JCAFC project, the long-term sustainability of this AFC project and the corresponding role of universities remain underexplored. The roles and responsibilities of intermediary organizations may evolve over time as ecosystems mature (Kivimaa et al., 2020). Hence, future studies could investigate the continuing role intermediary organizations may play as the AFC movement matures. Fourth, we did not collect the views from older adults who were not involved in JCAFC. It is plausible that older adults uninvolved in the initiative have different views on the role of universities in the construction of an AFC. Future research may consider conducting a comparative analysis to examine potential differences. Finally, this study solely focused on universities. As mentioned previously, other types of intermediary organizations may perform similar functions (e.g., think tanks) in advancing the AFC agenda. Future studies could expand its scope and consider these other players in society.

## Conclusion

The construction of an AFC is a complex, dynamic, and contextualized process and cannot materialize without the “buy-in” of all sectors of society. Using Hong Kong as a case example, this paper highlights the potential of leveraging credible and legitimate intermediary organizations, in our case, that of universities, to facilitate cross-sector collaboration, knowledge diffusion, interest group capacity-building, and mediation of divergent interests in a non-Western, quasi-democratic context to improve the overall vitality and effectiveness of the AFC ecosystem.

## Supplementary Material

igad016_suppl_Supplementary_Data
