# Supplementary material for: Universities as Intermediary Organizations: Catalyzing the Construction of an Age-Friendly City in Hong Kong
Source: Innov Aging. 2023 Feb 21;7(3):igad016. doi: 10.1093/geroni/igad016 (PMC10082544; doi:10.1093/geroni/igad016)
Supplement: igad016_suppl_Supplementary_Data [file igad016_suppl_supplementary_data.docx]

**Semi-structured interview guide**

By different interest groups

1. Local government
   - What are some motivations in participating in the project? What were your first thoughts when learning about this project?
   - What was your role in the JCAFC project?
   - Did you liaise with universities?
   - What did you think was the role of universities in this project? (HKU) What was useful? What can be improved?
   - How did you find your experience engaging in universities?
   - Without universities, what would be some anticipated challenges or benefits in implementation JCAFC?
   - What were particularly useful in engaging universities in similar community-based projects? How did engaging universities affected your own work in AFC?
   - What aspects can be further improved?
   - Now that the district has been admitted to WHO GNAFF, what future AFC endeavours will be initiated by government?
2. NPO representatives (social workers)
   - What are some motivations in participating in the project? What were your first thoughts when learning about this project?
   - What was your role in the JCAFC project?
   - Did you liaise with universities?
   - What did you think was the role of universities in this project? (HKU)
   - Without universities, what would be some anticipated challenges or benefits in implementation JCAFC?
   - What were particularly useful in engaging universities in similar community-based projects? How did engaging universities affected your own work in AFC?
   - What aspects can be further improved?
   - Now that the district has been admitted to WHO GNAFF, what future AFC endeavours will be initiated by your NGO?
3. Older adults
   - What are some motivations in participating in the project? What were your first thoughts when learning about this project?
   - What was your role in the JCAFC project?
     1. e.g. part of AFC community-based intervention, enrolled in ambassador program, attended theme-based AFC conferences
   - What did you think of these events?
   - What did you think was the role of universities in this project?
   - Without universities, what would be some anticipated challenges or benefits in implementation JCAFC? (or in their respective events or courses they have enrolled in)
   - What were particularly useful in engaging universities in similar community-based projects? How did engaging universities affected your own work in AFC?
   - What aspects can be further improved?
   - Now that the district has been admitted to WHO GNAFF, what future AFC endeavours will be initiated by yourself (older adults)?
